# Analysing trends and forecasting malaria epidemics in Madagascar using a sentinel surveillance network: a web-based application

**DOI:** 10.1186/s12936-017-1728-9

**Published:** 2017-02-13

**Authors:** Florian Girond, Laurence Randrianasolo, Lea Randriamampionona, Fanjasoa Rakotomanana, Milijaona Randrianarivelojosia, Maherisoa Ratsitorahina, Télesphore Yao Brou, Vincent Herbreteau, Morgan Mangeas, Sixte Zigiumugabe, Judith Hedje, Christophe Rogier, Patrice Piola

**Affiliations:** 1 Institut Pasteur de Madagascar, Antananarivo, Madagascar; 2 UMR 228 ESPACE-DEV (IRD, UAG, UM, UR), Station SEAS-OI, Saint-Pierre, 175 CD 26, 97414 L’Entre-Deux, Ile de la Réunion France; 3 Ministry of Health, Antananarivo, Madagascar; 4 Université de la Réunion, Saint-Denis, Ile de la Réunion France; 5 U.S. President’s Malaria Initiative, Antananarivo, Madagascar; 6 Centers for Disease Control and Prevention, Atlanta, GA USA; 7 Unité de recherche sur les maladies infectieuses et tropicales émergentes (URMITE), UMR 6236, Marseille, France; 8 Institute for Biomedical Research of the French Armed Forces (IRBA), Brétigny sur Orge, France

## Abstract

**Background:**

The use of a malaria early warning system (MEWS) to trigger prompt public health interventions is a key step in adding value to the epidemiological data routinely collected by sentinel surveillance systems.

**Methods:**

This study describes a system using various epidemic thresholds and a forecasting component with the support of new technologies to improve the performance of a sentinel MEWS. Malaria-related data from 21 sentinel sites collected by Short Message Service are automatically analysed to detect malaria trends and malaria outbreak alerts with automated feedback reports.

**Results:**

Roll Back Malaria partners can, through a user-friendly web-based tool, visualize potential outbreaks and generate a forecasting model. The system already demonstrated its ability to detect malaria outbreaks in Madagascar in 2014.

**Conclusion:**

This approach aims to maximize the usefulness of a sentinel surveillance system to predict and detect epidemics in limited-resource environments.

## Background

Early detection of outbreaks and rapid control actions are essential to prevent and contain the spread of infectious diseases to reduce morbidity and death. The implementation of an automated early warning system (EWS) is a key step in adding value to the epidemiological data routinely collected by surveillance systems to improve the timeliness of detection of diseases outbreaks. The World Health Organization (WHO) supports the strengthening of existing infectious disease surveillance systems by developing such EWSs [1]. Monitoring of the epidemic risk of malaria may integrate sequential and complementary components, such as an early detection system (EDS), an EWS and long-range forecasting (LRF) [1].

An increasing number of statistical methods for detecting changes in trends [2] have been developed, but there is not yet a single reference standard [3]. The absence of a gold standard in past epidemics and the lack of consensus [4] on outbreak characterization has serious operational implications and can become a stumbling block for EWS implementation.

**Fig. 1:**
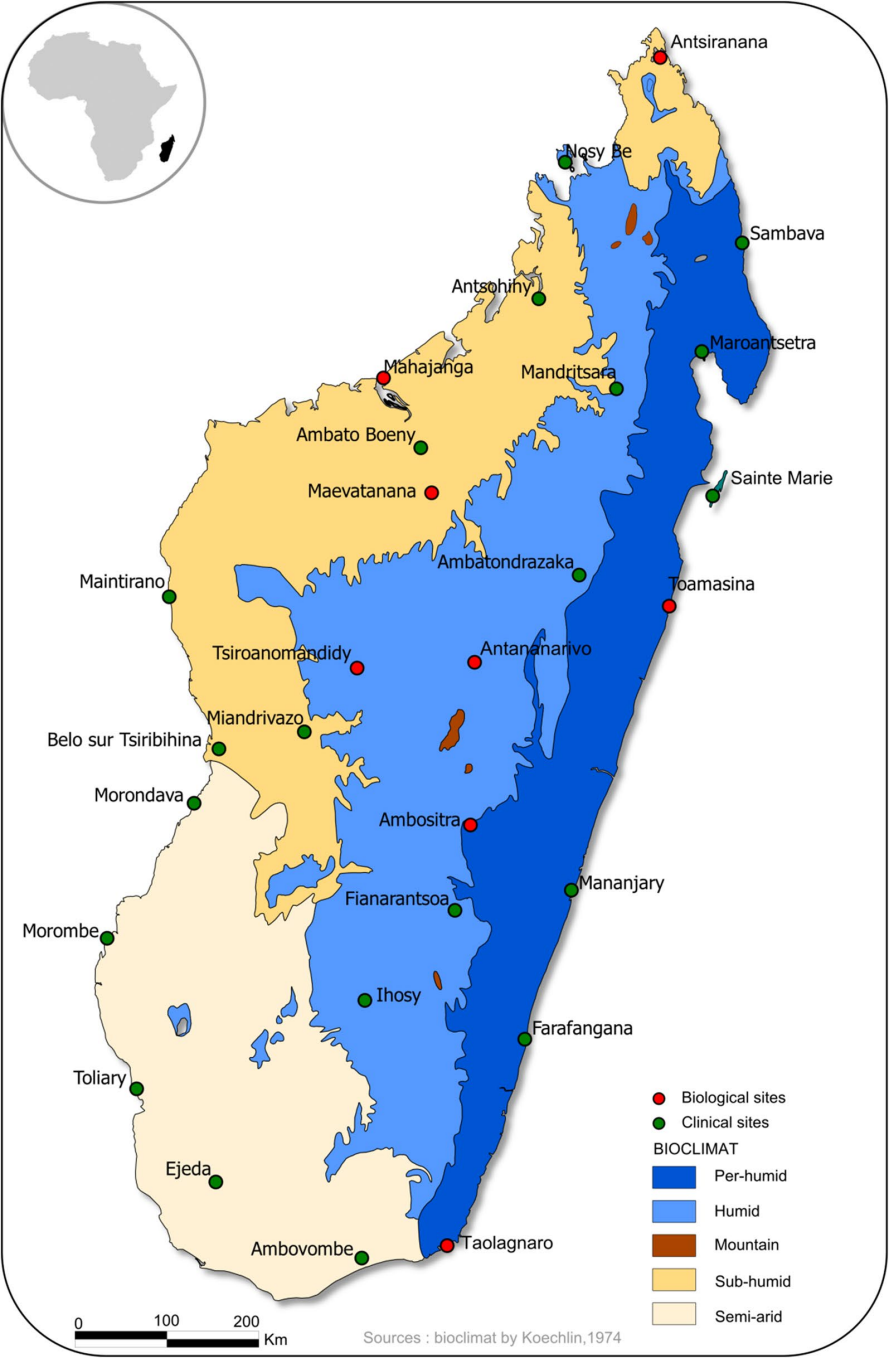
Surrounding climate and location of health centers participating in the sentinel surveillance system in Madagascar

Fever sentinel surveillance (FSS) using mobile health (mHealth) technology [5] has been implemented in Madagascar since 2007. This system records clinical data on several diseases, including malaria cases confirmed by a rapid diagnostic test (RDT), at 34 sites throughout the country (Fig. 1) [6, 7]. The development of methods providing lead time benefits before outbreaks is necessary to allow time for preparedness and to save lives [8]. By automating the analysis and visualization of outbreak detection, the need for time-consuming data preparation and analysis is reduced [9].

In Madagascar, malaria burden has decreased in recent decades, mainly due to successful malaria control interventions [10]. Nevertheless, an upsurge of malaria outbreaks in recent years has highlighted the need for a malaria EWS (MEWS) adapted to the Malagasy context. An innovative interactive MEWS with a web-based interface that includes standard, such as Cumulative-Sum or Mean +2 standard deviations [1, 11], and alternative outbreak detection methods could strengthen the national surveillance system. Recent open-source Internet technology allows processing of surveillance data and application of outbreak detection algorithms with automatic and interactive graphical feedback [12]. Thus, current web-based technologies allow user-friendly assessments of an outbreak hypothesis with model comparisons using prospective surveillance data as well as retrospective descriptions of the effects of malaria epidemic. An integrated system for real-time detection and forecasting could also be a pathway for the dissemination and communication of results.

Here, this study describes a system with intertwining of new electronic health (e-Health) technology (i) to assess the benefits of a MEWS including not only early detection but also forecasting based on a sentinel surveillance system, (ii) to maximize the potential of the sentinel surveillance system by innovative but simple explorations of population health data, and (iii) to provide practical examples and suggestions for use in other systems or settings.

## Methods

### Fever sentinel surveillance network

The Institut Pasteur de Madagascar (IPM) and the Malagasy Ministry of Health (MoH) implemented a sentinel surveillance system based on primary health care centers (PHCCs); this system expanded from 13 sites in 2007 to 34 sites in 2011. Expansion was intended to improve geographic coverage and representativeness and to make it possible to monitor epidemiologic trends in different climate zones [6] (Fig. 1). Sentinel sites include the presence of at least two general practitioners and a mobile phone network available. Participation is entirely voluntary. Sentinel general practitioners (SGPs) serving on a gratuitous, voluntary basis are the backbone of the system, which currently covers about 8% of the Malagasy population. Supervision of sentinel sites is performed twice a year, either by the team of the medical inspector or by the IPM team and the central level of the Ministry. There is an evaluation of the quality of the rapid diagnostic test used by the sentinel sites and a verification of the quality of the data collected during the supervision. The system monitors several potential epidemic diseases: malaria, influenza-like illnesses, suspected arboviral infections and diarrhoeal syndromes. Per national policies, every febrile patient is tested for malaria infection with an RDT. Data are aggregated and reported daily by short message service (SMS). The data are then automatically stored in a PostgreSQL database hosted on a dedicated server at IPM. The data received by SMS include: sentinel site code, date of data collection, total number of outpatient consultations, total number of confirmed malaria cases, total number of ILI cases, total number of dengue-like cases, total number of diarrhoea cases, and the number of consultations by age group. The age groups were those commonly used by the Ministry of Health in Madagascar: less than 1 year, 1–4 years, 5–14 years, 15–24 years, 25 years and over. The reporting system, which is based on mHealth technology, has been improved using Android smartphones and dedicated data entry forms.

### Statistical detection methods

There are several different ways to define epidemic alert thresholds, and the three most commonly used methods have been included in the surveillance system: (i) Mean + 2 standard deviation (Mean + 2SD) [1]. The method is based on the weekly mean calculated from previous 5 years plus 2 standard deviations where “Epidemic years” must be excluded; (ii) Cumulative SUM + 2 standard deviations (C-SUM + 2SD) [1]. This method is a derivative of the mean + 2 standard deviations. To improve specificity, expected number of cases used the average for 3 weeks (including the previous and following weeks) [4] during the previous 5 years plus two standard deviations [13, 14]; and (iii) the weekly slope [15]. The method is defined as a doubling of cases during 3 consecutive weeks. Weekly slope is included in core policy documents [15] from the Malagasy MOH. A fourth percentile-based method has been specifically developed by IPM using a threshold defined as weekly malaria cases exceeding the 90th percentile value. The percentile value is not seasonal-dependent and calculated over the whole chronological series of a site.

For these four methods, an alert is triggered if the defined threshold is exceeded for the three previous consecutive weeks 3 to increase the specificity of the alert system for any given threshold [16]. These four algorithms were applied on the FSS dataset to determine the operability of signals and to assess the algorithms’ usefulness in the identification of outbreaks. To reduce noise in detected signals, 13 sites with a maximum weekly number of malaria cases lower than 10 were excluded.

Each detection method was applied to each sentinel site over the 52 weeks from 2014-05-26 to 2015-05-18, representing the last complete cycle of malaria seasons, such as low season (LS), moderate season (MS) and high season (HS). All historical datasets since the setting up of sentinel surveillance in 2007, excluding the year (52 weeks) being tested, were used to define the baseline for the Mean + 2 SD and C-SUM + 2 SD methods [16]. “Epidemic years” were not excluded from the base years because there is no standardized method to define them retrospectively and because the MoH has not officially reported any epidemics.

### Forecasting model

Forecasting may rely on several techniques related to statistics and mathematical modeling or machine-learning methods [17]. The forecasting method used on sentinel dataset is based on a statistical method known as Seasonal Auto-Regressive Integrated Moving Average (SARIMA) [18], with use of external regressor variables (SARIMAX) [19–21] including satellite weather data and information on transmission-reducing interventions. SARIMA(X) models are designed to account for serial autocorrelation in seasonal time series.

Satellite weather data related to changes in malaria prevalence such as temperature, rainfall and Normalized Difference Vegetation Index (NDVI) [17] provided by the International Research Institute for Climate and Society (IRI) through a web server [22] are routinely and automatically acquired by the surveillance system. Historical data up to April 2007 are also downloaded. The data are processed to match epidemiological weeks and are stored in a PostgreSQL database.

Time–space data on Malaria Control Interventions (MCIs) were obtained from national (National Malaria Control Programme) and international (President’s Malaria Initiative) agencies in charge of malaria control in Madagascar. Indoor Residual Spraying (IRS) data have been available only since 2008. Two LLIN mass distribution campaigns were implemented in Madagascar at the end of 2009 and 2012 (coverage ranges from 80 to 94%). Data on LLIN distribution were available at district level on a weekly basis and encoded in the database as a binary variable: weekly absence or presence of distribution.

### Retrospective analysis

A retrospective analysis was performed on sentinel sites for which a malaria alert was detected by this system and subsequently confirmed by an epidemiological investigation. The SARIMA(X) model was selected using the forecast package [23], which is available for the R programming environment [24]. Model selection was automated using the auto.arima function, which performs a stepwise regression on the data and selects the best model based on the Akaike Information Criterion (AIC). The time series were log transformed to induce constant variance. Rainfall and temperature were log transformed and lagged from 0 to 8 weeks to account for the delayed effects of weather on malaria infections [25]. The variable representing interventions against malaria was defined as the time elapsed in weeks since the last intervention and was categorized as (i) less than 1 year (≤52 weeks, reference) (ii) >52 weeks and ≤104 weeks, (iii) more than 2 year (>104 weeks) [Girond et al. pers. comm.]. To select the most pertinent predictors, both forward and backward stepwise methods were used [26]. The entire dataset and all associated variables were used to train the model.

### Prospective analysis

**Fig. 2:**
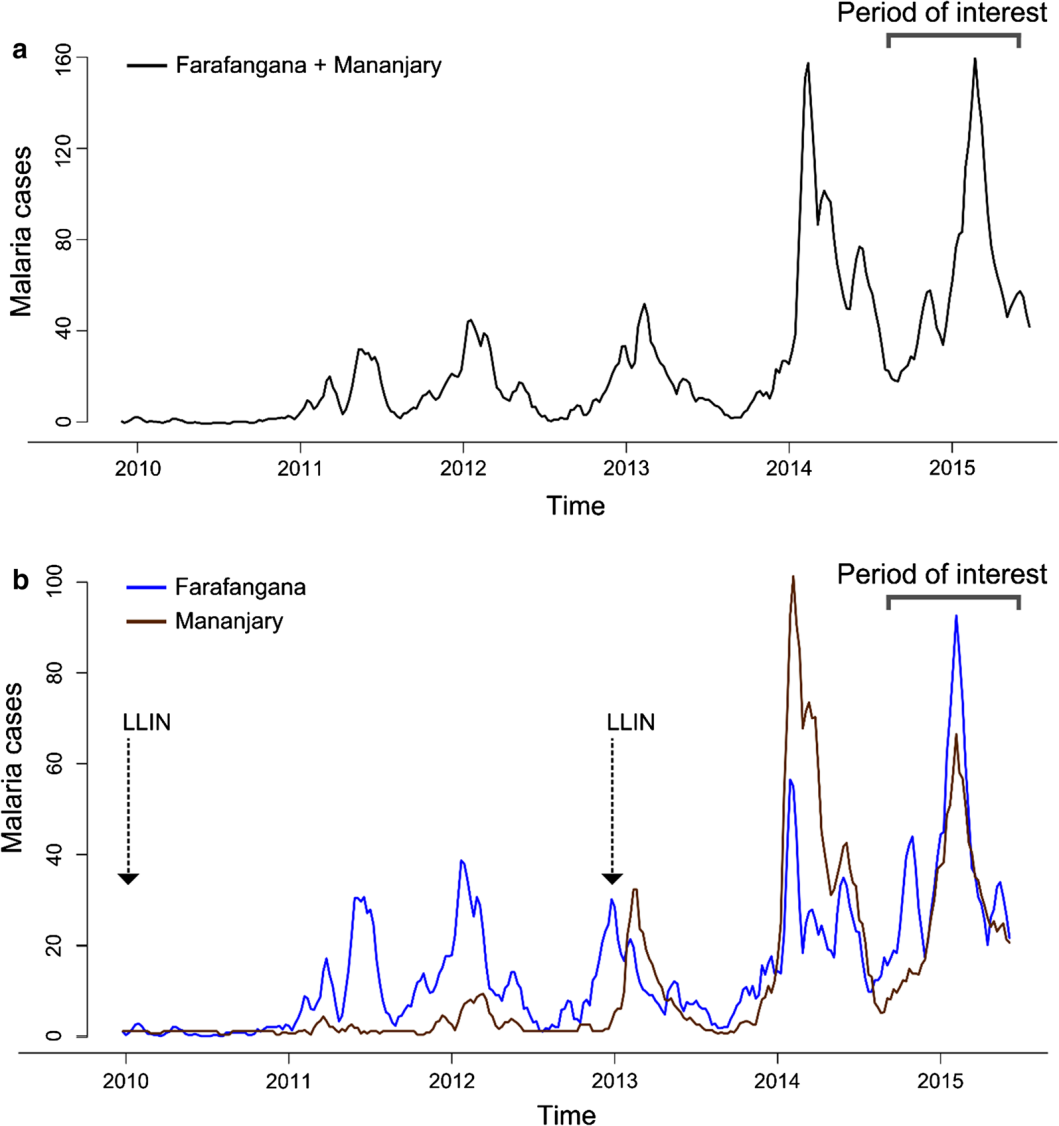
**a** Historical time series of malaria cases recorded at both Farafangana and Mananjary, **b** historical time series of weekly malaria cases recorded at Farafangana and Mananjary with mass distribution campaigns of LLINs

Retrospective analysis establishes associations between outbreaks and the external regressor variables [27]. To investigate the predictive accuracy of the SARIMAX model, a prospective analysis was performed. The Farafangana and Mananjary sites were merged into one single time series to improve the statistical power of the SARIMA model (Fig. 2a). These sentinel sites present similar characteristics in their geographical position (southeastern coast) and bioclimates (perhumid), with concomitant LLIN distribution (end of 2010 and 2012). The last two peaks of malaria cases for each site were recorded during the same week (i.e., on 2014-01-13 and 2015-01-05) (Fig. 2b). External regressors such as log rainfall and mass distribution campaigns of LLINs are included in the forecasting model as predictors.

The model was fitted using data from 2009-12-21 to 2015-02-16. The last 52 weeks of the time series were withheld from model fitting and used to make a one-step-ahead forecast. The simulation proceeded by iteratively adding a new week of data, training a new model based on the updated data, and predicting the number of malaria cases for the following weeks (n = 231 for model development, n = 52 for external validation). The pre-dic-tive per-for-mance of the SARIMAX model was estimated using a confusion matrix [28], showing the proportions of predicted outbreak and detected outbreak based on the percentile-based method over the 52 weeks.

### Website

**Fig. 3:**
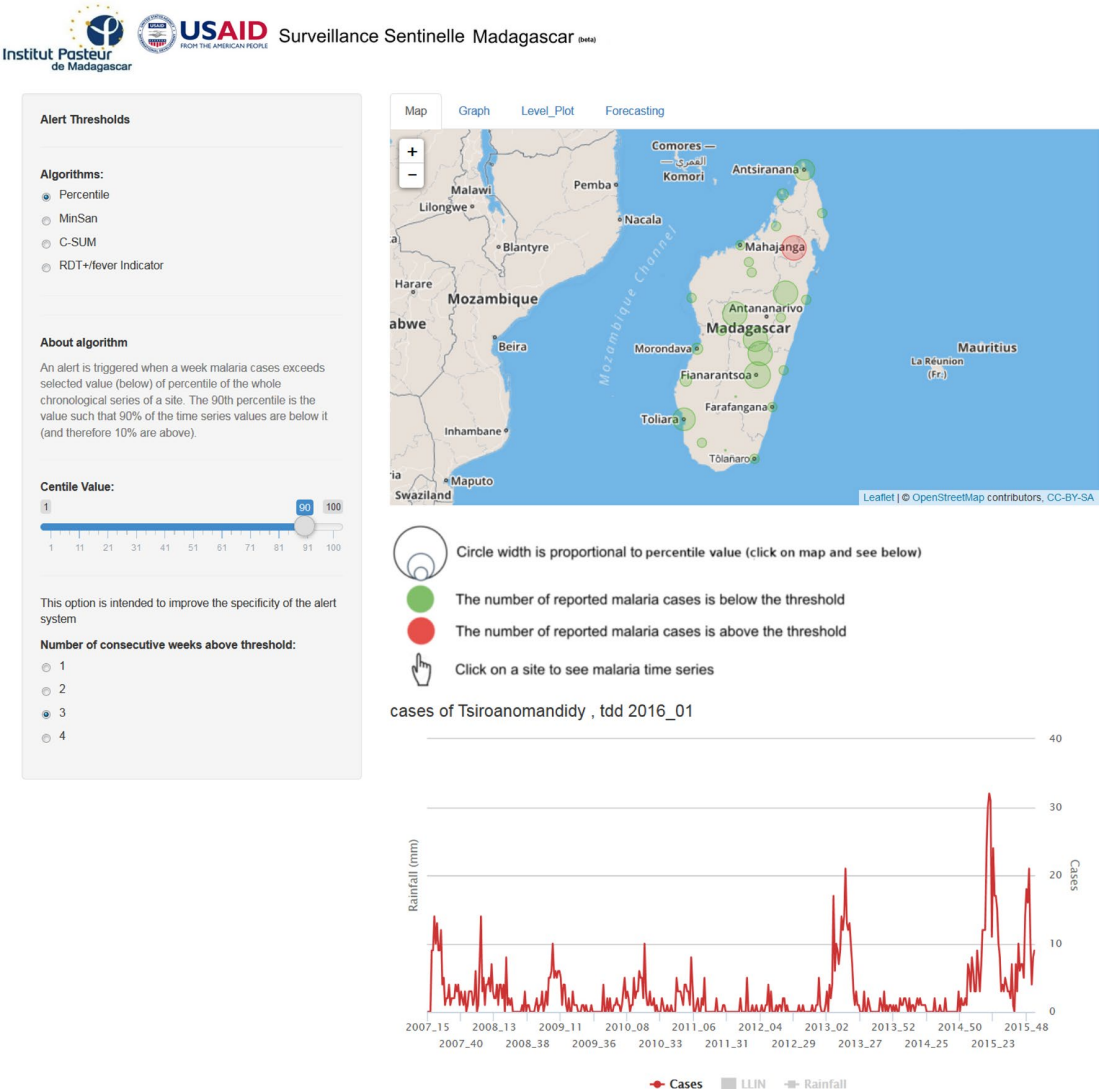
Screen print of the web-based MEWS, accessible through a user-friendly interface

A free, open-source and fully automated and interactive web-based interface has been developed using the Shiny package for the R programming language [12] (Fig. 3).

## Results

### Early detection

**Fig. 4:**
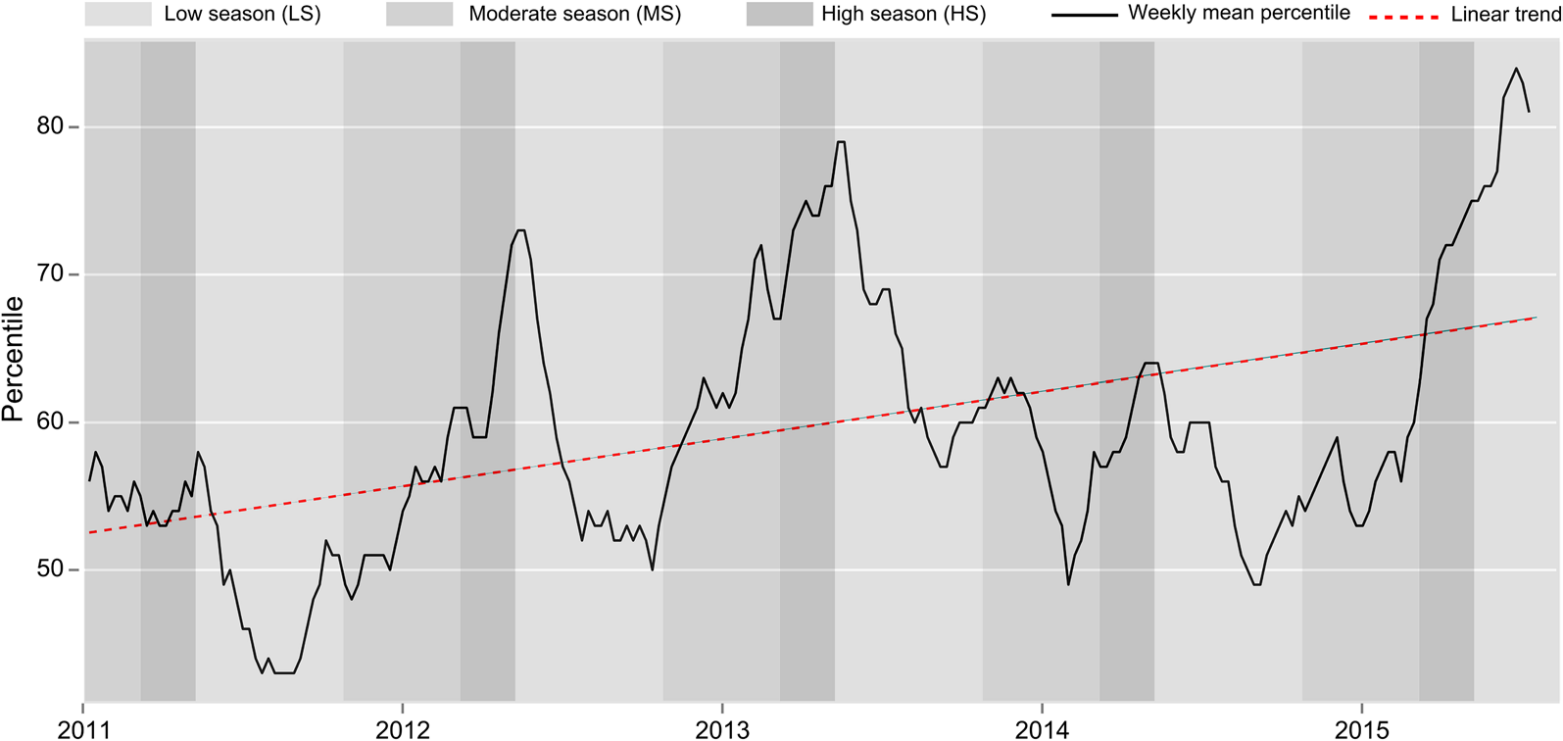
Time series of the average of percentile values of weekly malaria cases recorded over the 21 sentinel sites since 2011 with linear trend

From April 2007 to May 2015, 34 578 malaria cases confirmed by an RDT have been recorded at the 21 sites included in this study. The number of recorded malaria cases has steadily increased since the FSS completed its implementation in 2011. Based on the data, the following linear regression line was determined: Y = –53.9 + 0.0568 X, where X is the time in weeks and Y is the mean of the percentile values of the weekly number of malaria cases across the 21 sites since 2011 (R-squared linear = 0.184) (Fig. 4).

**Fig. 5:**
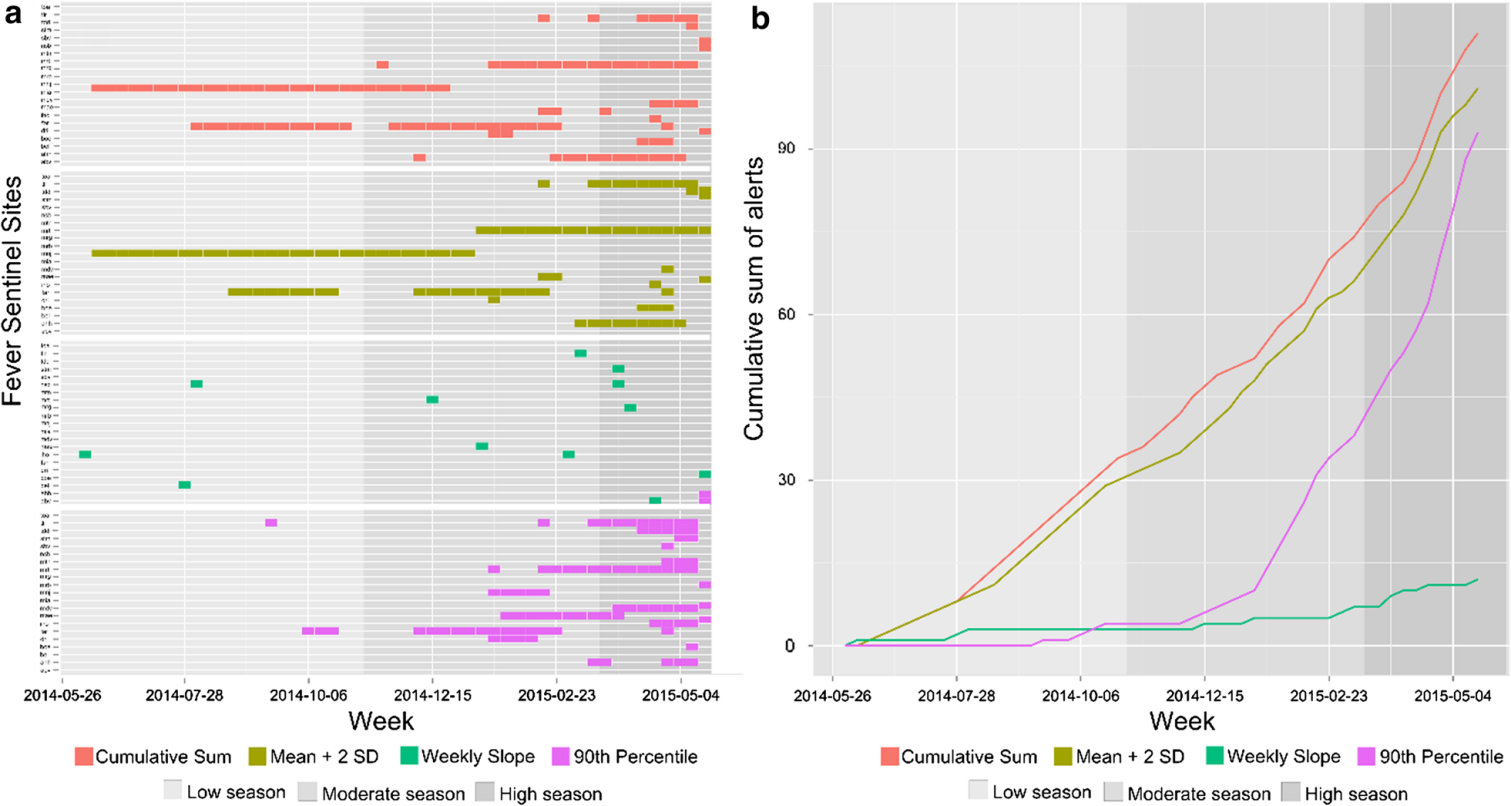
**a** Time-series of outbreak alerts for the 21 sentinel sites. The results of outbreak detection techniques are shown for the Mean + 2 Sd, C-SUM, MoH and percentile-based techniques, **b** Time series of cumulative sum of outbreak by outbreak detection methods. The results of outbreak detection techniques are shown for the Mean + 2 SD, C-SUM, MoH and percentile-based techniques

The comparison of the outbreak characteristics at each sentinel site over 52 weeks showed high heterogeneity in signals across both time and space, such as the alert incidence, the alert duration and the number of alerts triggered. The total number of signals detected differed across outbreak detection methods. The number of signals generated by C-SUM + 2 SD, Mean + 2 SD and the percentile-based method, was 111, 102, and 93 alerts, respectively. During the same study period, 12 alerts were triggered by the Weekly slope method (Fig. 5a).

An incidence analysis of signals showed inter- and intra-variability for the detection methods. The frequency of signals was roughly equivalent across transmission periods for C-SUM + 2 SD and Mean + 2 SD. The C-SUM + 2 SD method generated a stable incidence of alerts, with 35, 42 and 34 alerts in the LS, MS and HS respectively. The Mean + 2 SD method was also constant, with 31, 37 and 34 alerts, respectively. The frequency of alerts was progressive for the MOH method across the transmission periods, but the numbers of alerts were low, with 3, 4, and 5 for LS, MS and HS, respectively. Alert frequencies increased gradually across the three periods of transmission with the percentile-based method, with 4 alerts triggered during LS, followed by 38 alerts during MS and 51 alerts during HS. The frequency of alerts started its sharp rise in the middle of MS (Fig. 5b).

The variability of the duration of the detected signals was noteworthy across detection methods. The range of durations of detected signals for Mean + 2 SD and C-SUM + 2 SD varies from 1 to 31 weeks and 29 weeks, respectively. The duration of detected signals by percentile-based method ranges from 1 to 13 weeks. The maximum duration of detected signals for the MoH method did not exceed 1 week (Fig. 5a).

### Outbreak detection

An outbreak in the southeastern part of the country for the sentinel site of Farafangana (Fig. 1) on 2014-10-06 has been detected using the percentile-based method. This detected signal indicated that a historical level of malaria cases was reached 6 weeks before the moderate transmission period and 6 months before the high transmission period.

### Retrospective SARIMA evaluation

The SARIMAX model [1, 4] (1, 0, 1) had the lowest AIC value (77.89) for this dataset and therefore was the best-fit model, with root-mean-square deviation (RMSE) = 0.26 and mean absolute scaled error (MASE) = 0.15. Thus, to conclude, the SARIMA [1, 4] (1, 0, 1) 52 model fit the data well.

### Prospective SARIMA evaluation

**Fig. 6:**
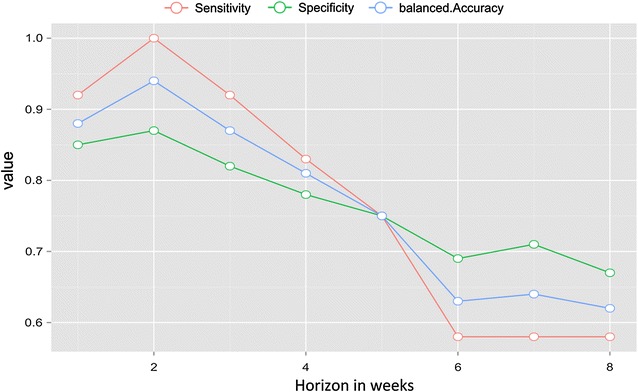
Forecast accuracy measures for different forecast horizon (h)

During the period of interest of 52 weeks from 2014-05-26 to 2015-05-18 (Fig. 2b), 16 weeks were defined as an outbreak. Malaria outbreaks were predicted with a sensitivity of 83% and a specificity of 78% up to 4 weeks in advance (accuracy of 0.80%, 95% CI 0.66, 0.90). Beyond the 5 weeks of horizon forecasting, the accuracy fell to below 75% sensitivity and specificity (Fig. 6).

### Mhealth and website

The reporting system based on mHealth technology, is using the Android operating system smartphones. This new open-source technology runs through a dedicated application developed by IPM, involving handheld data entry in the national language, a feedback report with automated analysis via charts and maps, and an edutainment-based learning solution. No Internet is required, which avoids the need to cope with patchy Internet coverage; the Android application generates all outgoing SMS messages, which are streamlined into the central surveillance server, and decrypts the “feedback SMS” generated by the surveillance server.

The operational web-based surveillance system includes both an EDS and a forecasting model. The website is accessible to Roll Back Malaria partners [29]. Epidemic threshold detection algorithms are integrated into the website and applied to the sentinel dataset in real time (Fig. 3). The selected detection methods can be easily modified by changing the number of years in the baseline dataset (i), the number of standard deviations (ii), the slope value (iii), the percentile value (iv) and the number of weeks above the threshold (v) by intuitive pick-and-click functionality. The results are instantly displayed both on interactive charts and maps. Users can superimpose additional data such as temperature, rainfall, and NDVI data from satellite Earth observation and also malaria control interventions (LLIN use and IRS).

## Discussion

**Fig. 7:**
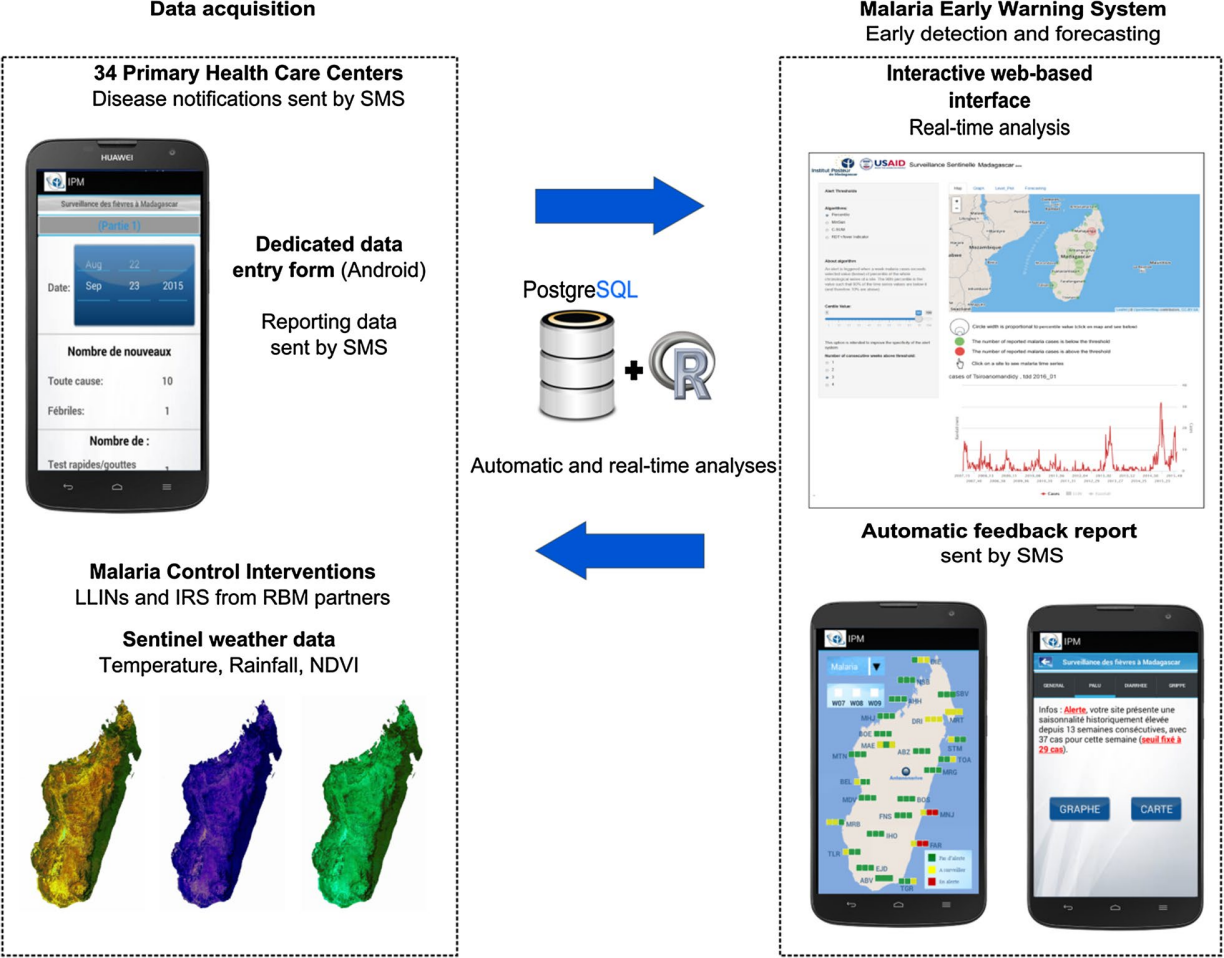
Schematic representation of the system architecture

The development of an automated and graphical MEWS is a promising approach to enhancing early outbreak detection and rapid response capacity in Madagascar. Specifically, a MEWS has been integrated into the routine processes of outbreak monitoring and response by all stakeholders in Madagascar. This integration involved intertwining of new technologies, such as satellite observation of environmental conditions and use of geographical information systems, mHealth components and interactive web analytical tools (Fig. 7). The technology platform has been designed to help the health sector to interpret signals in surveillance data. Indeed, the statistical methods within an automated system cannot be used to confirm an epidemic requiring action but could be used to extract significant changes hidden in routine tables of sentinel weekly data and to allow the epidemiologist to focus on specific data points. The system involves statistical methods adapted to the surveillance context to respond as best possible to the operational objectives in resources limited settings. Recommended detection methods (i.e., from the WHO and the Centers for Disease Control and Prevention (CDC) are too sensitive to be used in such process in which a high number of “false” alerts could be irrelevant to the malaria situation. These methods are ultimately too restrictive in their conditions of use (minimum of a 5-year dataset, exclusion of epidemic years) and then are inappropriate for existing FSS data because of inadequacy in their prerequisites. Even with high data quality, the application of these approaches appears not to be adaptable to areas characterized by not only large variations related to the level and seasonality of transmission but also a clear trend in malaria cases (Fig. 4) [13, 30]. For this reason, the project has moved toward the use of an alternative and less restrictive method in which an outbreak is defined as the 90th percentile of the whole chronological series of a site during three consecutive weeks. The 90% threshold could be modified to adapt to previous epidemics or new targets in a context of successful malaria control. The absence of baseline construction based on historical data in the calculation method allows retrospective analysis of a whole-time series in which epidemic years have not been arbitrarily excluded. Signals detected based on percentiles become easily interpretable for public health activities because they are representative of the most prominent historical events recorded.

This sentinel surveillance coupled with a technology platform has yielded positive results, detecting the 2014-10-06 outbreak in the southeastern part of the country. The web-based surveillance system, with automated analysis and timely output, allowed real-time monitoring and communication with RBM partners about this malaria event. The high number of malaria cases reported, and the assumption of the existence of a plasmodium reservoir preceding the rainy season together with limited access to artemisinin-based combination therapy (ACT) in the whole area suggested a worsening malaria situation the following weeks. The affected local public health jurisdiction concomitantly alerted the MoH about excess mortality and morbidity beyond their response capabilities [31], and this outbreak was subsequently confirmed by an epidemiological investigation.

The use of methods based on several consecutive weeks above the threshold, with the aim of improving the methods’ specificity, accordingly reduced the methods’ ability to detect incipient outbreaks at the earliest stages. An EDS has to be strengthened by a forecasting method to provide lead time benefits [8]. The malaria outbreak on the eastern coast was predicted with a sensitivity of 83% and a specificity of 78% up to 4 weeks in advance (accuracy of 0.80%, 95% CI [0.66, 0.90]). Nevertheless, the model predicted a threshold overrun, but the stochastic behaviour of epidemics limited the prediction of the amplitude. The system can give timely alerts for epidemic control, even if it is unable to provide very accurate predictions of malaria case numbers. The improved lead time of an EDS, however, comes at the expense of a degree of accuracy [32].

The MEWS is accessible through a user-friendly web-based interface [29] for both internal use and use by external organizations and donors. This MEWS allows rapid dissemination, interpretation and subsequent action to control any suspected outbreak. Recent open-source technology also allows for the development and improvement of an interactive web-based interface with dedicated analysis and visualization output. Furthermore, based on R language (coupled with Shiny package), its growing popularity in the scientific community makes this technological platform easily modifiable and maintainable and also transferable.

The detection and reporting methods for malaria cases of the sentinel surveillance system remained unchanged since its implementation. The increasing trend observed from 2011 to 2015 (Fig. 4) reflects the malaria situation at national level over the same period [33]. The outbreak thresholds in this analysis were defined based on the absolute number of malaria cases due to the lack of population denominators to calculate the malaria incidence for health facilities. The population size in the catchment area of each facility (denominator) is also unavailable and varies with the availability of health care from the private and informal sectors. Forecasting models and surveillance systems should be improved through the integration of additional covariables, such as the availability of ACT. The resurgence of malaria across most of Madagascar in 2014 occurred in the context of nearly generalized ACT stock-outs. Furthermore, the inclusion of individual data in the surveillance system would allow enhanced description of malaria transmission (i.e., description of the most vulnerable age groups). Such a system might also be reinforced by integration of transmission-reducing interventions on a smaller scale across both time and space. Staffs from the Ministry of Health were involved in the project through regular working group meetings and MoH medical epidemiologists were permanently detached in our team to ensure a constant transfer of knowledge and experience. The Health Monitoring and Disease Surveillance were very supportive of this sentinel project and used several of its successful components to improve their nationwide surveillance system. There are indeed challenges in extending an electronic based surveillance system to an entire country, although it is admitted that the current paper-based surveillance system does not allow a prompt analysis of trends to detect emerging epidemics. A progressive scaling-up of e-surveillance to health centres using affordable technologies is deemed reasonable and efficient, and is currently being promoted by WHO as a way forward. This technology-based approach to surveillance has a great potential for real-time evaluation of malaria control interventions at both the national and the regional levels.

## Conclusion

The authors describe an automated malaria outbreak detection system using percentile-based statistical detection method that uses data electronically collected in Madagascar by FSS. The system assesses data as soon as they are made available and disseminates the information by means of the Internet and smartphone to all involved health professionals to help in the rapid interpretation and subsequent action to control any suspected malaria outbreak.

Much still needs to be done and efforts are now focusing on the expansion of the surveillance system with the aim of a progressive and realistic “strengthening” to improve outbreak detection and forecasting system to malaria elimination. This approach, entirely based on free and open-source technology, should also benefit other initiatives aimed at improving surveillance data management in other health care facilities and provides a demonstration project for improving existing systems in Africa.
